# Seven reasons why threatened species managers must consider genetic health

**DOI:** 10.1093/biosci/biag046

**Published:** 2026-05-14

**Authors:** Nicholas J Bail, Conrad J Hoskin, Harry B Hines, Richard Frankham, Megan Higgie

**Affiliations:** College of Science and Engineering, James Cook University, Building 142, 1 James Cook Drive, Townsville, QLD 4811, Australia; Centre for Tropical Bioinformatics and Molecular Biology, James Cook University, Townsville, QLD 4811, Australia; College of Science and Engineering, James Cook University, Building 142, 1 James Cook Drive, Townsville, QLD 4811, Australia; Queensland Parks and Wildlife Service and Partnerships, Department of the Environment, Tourism, Science and Innovation, PO Box 64, Brisbane, QLD 4070, Australia; Adjunct Research Associate, Biodiversity and Geosciences Program, Queensland Museum, South Brisbane, QLD 4101, Australia; School of Natural Sciences, Macquarie University, Sydney, NSW 2109, Australia; Australian Museum, 1 William Street, Sydney, NSW 2010, Australia; College of Science and Engineering, James Cook University, Building 142, 1 James Cook Drive, Townsville, QLD 4811, Australia; Centre for Tropical Bioinformatics and Molecular Biology, James Cook University, Townsville, QLD 4811, Australia

**Keywords:** genetic management, conservation, genetic diversity, inbreeding, augmented gene flow

## Abstract

Genetic management is well established as an essential component of threatened species management. In small populations, deteriorating genetic health—such as increases in inbreeding and losses of genetic diversity—becomes an important cause of extinction. Cost-effective genetic management practices exist, but a lack of awareness limits widespread application. We provide seven reasons threatened species managers must consider genetic health, each of which can be read in under 5 min. These reasons are intended to be a synthesis for a broader audience than is generally reached by specialist scientific literature. Consequently, we aim to empower threatened species’ managers, agencies, and policymakers to recognize the relevance of genetic health to the species under their responsibility and to actively seek the support of conservation genetics experts.

Globally, extinctions are occurring at an alarming frequency—far above the long-term natural background extinction rate. At least 680 vertebrate species have become extinct due to human actions since 1500, and an estimated one million species now face extinction (IPBES 2019). A recent estimate puts the cost of preventing extinction for only 99 priority Australian species at approximately USD$10.06 billion per year for 30 years, comparable to 1% of Australia’s GDP per year (Ward et al. 2025). In addition, conservation around the world is severely underfunded, hampering conservation efforts for most threatened species (Waldron et al. 2013, Wintle et al. 2019, Eberhard et al. 2022). In this resource-poor context, it is necessary to identify the main threats to species and to implement the most appropriate, cost-effective, and feasible conservation strategies to address them (Ward et al. 2021).

The most serious threats to species are commonly identified as environmental factors: habitat loss, disease, invasive species, and climate change (Ward et al. 2021, Hogue and Breon 2022). However, once a species is already threatened, genetic health can become one of the most critical considerations for species’ survival or extinction (Ralls et al. 2018, Kardos et al. 2021). “Genetic health” refers to the genetic characteristics of a population that directly affect extinction risk and survival. Box 1 introduces, with definitions, the five key metrics of “genetic health.” It is generally overlooked that poor genetic health can become one of the most threatening processes affecting wild populations (Ralls et al. 2018, Kardos et al. 2021). There is also a perception that genetic issues are difficult and expensive to fix (Taylor et al. 2017, Cook and Sgro 2018). However, as we outline, strategies for genetic management are cost-effective, with large benefits for individual fitness, population growth, and species resilience (Frankham 2015, Weeks et al. 2017, Fitzpatrick et al. 2020).

BOX 1: **Definitions for the five key metrics of genetic health.**

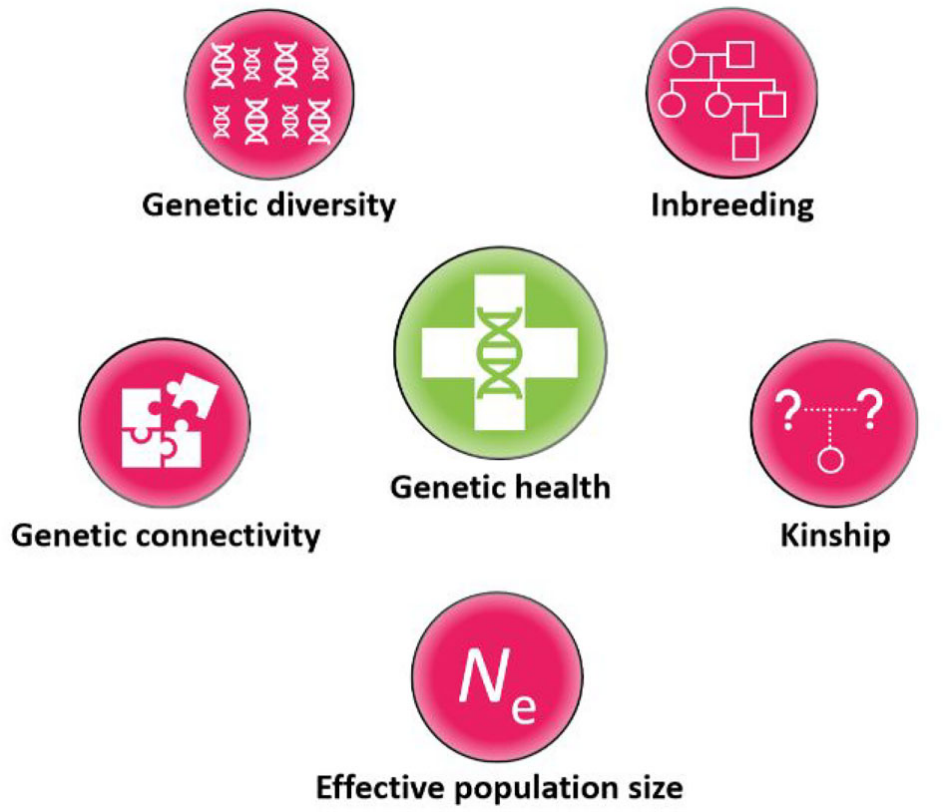

**Table:** 

Metric	Importance to genetic health
Genetic diversity	*High genetic diversity contributes to good genetic health*. Genetic diversity is the extent of genetic variation in an individual, population, or species (e.g., heterozygosity or allelic richness). It can be rapidly lost in small, isolated populations. Without genetic diversity, populations have a reduced ability to adapt to other threats, and an increased extinction risk (Frankham 2005, 2015, Allendorf et al. 2022). For basic genetic health assessments, we emphasize the value of measuring neutral genetic diversity over functional genetic diversity because of the difficulty in measuring and identifying which functions are important now or in the future (Kardos et al. 2021).
Inbreeding	*Low levels of inbreeding contribute to good genetic health*. Inbreeding is the mating of genetically related individuals, which leads to their offspring inheriting duplicate copies of harmful alleles that are usually hidden in outbred populations (i.e., populations where mating occurs between unrelated individuals). The resulting loss of fitness is known as inbreeding depression and is usually unavoidable in small, isolated populations. Inbreeding depression has direct consequences for both individual fitness and increases a populations’ extinction risk (Frankham et al. 2010).
Kinship	*Low mean kinship contributes to good genetic health*. Kinship is very similar to the idea of genetic relatedness. In the context of genetic health, kinship is considered one of the best ways to measure genetic similarity between individuals, between populations, and throughout populations (Frankham et al. 2017). When crossing individuals or populations, kinship allows the risks of inbreeding and gains in genetic diversity to be predicted (i.e., higher kinship between breeding individuals means inbreeding will be higher and little new genetic diversity will be contributed (Frankham et al. 2017).
Genetic connectivity	*High genetic connectivity contributes to good genetic health*. Genetic connectivity is the ability for individuals or their gametes to move between populations and contribute genetically to those populations. Isolated populations have low or no connectivity and are at higher risk of losses of genetic diversity and increases in inbreeding. Conversely, increased genetic connectivity can recover genetic diversity and reduce inbreeding (Frankham 2022). Understanding genetic connectivity is often a first step in a genetic health assessment because it is required to define the populations and population structure in the assessment (Frankham et al. 2017).
Effective population size (*N*_e_)	*High effective population size contributes to good genetic health*. This is a number representing the size of an idealized population that would account for the observed rate of loss of genetic diversity (or the rate of increase of inbreeding) in a real population (Frankham et al. 2010, Waples 2025). A common misconception is that *N*_e_ is “the number of adults who contribute to the next generation” (Waples 2022). Instead, several characteristics of a population contribute to the rate of loss of genetic diversity and thus determine *N*_e_ (e.g., census size, variation in population size over generations, variation in reproductive success, sex ratio, etc.). As such, effective population size is far more important to genetic health than census size (“*N*_c_”; Frankham 1995, Waples 2022, 2025). The ratio between the two varies widely (Waples et al. 2013), but effective population size estimates generally average about one-tenth of the census population size (Frankham 2021).

Despite the long-known consequences of poor genetic health for threatened species, the concept is often left out of conservation policy, plans, and activities. This is frequently referred to as the “conservation genetics gap” (Klütsch and Laikre 2021). As an example of this gap, only 7% of species’ recovery plans across Australia, Europe, and the United States mentioned inbreeding, despite its role in species’ extinction risk (Pierson et al. 2016). Similarly, Cook and Sgro (2017) found that less than half of Australian conservation policy documents mentioned the basic concept of genetic diversity. Conservation managers generally agree that, despite having a positive view of genetics, they rarely integrate genetic concepts into conservation, let alone directly address genetic issues (Taylor et al. 2017, Cook and Sgro 2018). Based on surveys of both conservation managers and scientists, the key reasons for this gap seem to be underfunding of conservation, the need for detailed genetic knowledge, rapid advances in genetic methods, and ineffective communication between managers and scientists. Consequently, many managers still report that the benefits and applicability of genetic management need to be demonstrated to them (Taylor et al. 2017, Cook and Sgro 2018, Taft et al. 2020).

However, the responsibility for genetic management is not just on conservation managers. Many aspects of threatened species management other than conservation genetics depend on scientists or other specialists to bring expert knowledge and keep abreast of advances; for example, pathology and epidemiology, population viability analysis, species distribution modeling, invasive species management, and habitat restoration (e.g., Cook et al. 2013, Walsh et al. 2015, Cook and Sgro 2017, Jellinek et al. 2021). Conservation managers are not expected to be experts in any of these areas. Instead, their role is to understand the importance of each topic as well as some of the fundamental concepts so that they can navigate the complex logistics of decision-making, stakeholder engagement, and implementing the resulting conservation actions. Strong collaboration between conservation managers and scientists is therefore a prerequisite for effective conservation actions (Cook et al. 2013, Shaw et al. 2024).

Fortunately, there are some excellent examples of genetic management in conservation implemented through collaboration between conservation managers and scientists (Garner et al. 2016, Shaw et al. 2024). This includes African Lion (*Panthera leo*; Miller et al. 2020); Brown’s Banksia (*Banksia brownii*; Dillon et al. 2023); California Condor (*Gymnogyps californianus*; Ralls and Ballou 2004); Eurasian Beaver (*Castor fiber*; Taylor et al. 2024); Giant Panda (*Ailuropoda melanoleuca*; Lan et al. 2024); Golden Lion Tamarin (*Leontopithecus rosalia*; Magro Moraes et al. 2023); Helmeted Honeyeater (*Lichenostomus melanops*; Pavlova et al. 2023); and Macquarie Perch (*Macquaria australasica*; Pavlova et al. 2024). Despite these great cases, the window of opportunity is quickly closing for the many threatened species on the brink of extinction, and action is needed in the next few years (Ralls et al. 2018, IPBES 2019).

## Aims of this paper

Drawing on compelling scientific evidence, we aim to provide conservation managers with easy-to-understand reasons to begin genetic management. The genetic management options we recommend often involve simple, cost-effective remedies to serious genetic problems. Although many conservation managers may seek further knowledge, our goal is that conservation managers reading this will simply recognize the importance of genetic health and the efficacy of genetic management at all levels of threatened species management. And consequently, that conservation managers are encouraged to collaborate with scientists to implement genetic management.

This paper focuses on genetic health (defined in box 1), as differentiated from other aspects of conservation genetics such as the important use of genetics to delineate species prior to undertaking genetic health assessments (e.g., using phylogenetics; Allendorf 2017, Frankham et al. 2017). In addition, this paper is a synthesis rather than a full review, so we include reference to a sample of more detailed resources suited to multiple levels of application and experience, enabling managers to find information that is most relevant to their context.

Here, we outline seven reasons why genetic health must receive greater consideration by conservation managers (listed in table 1). Reason 1 explains the ubiquity of poor genetic health in threatened species. Building on this, Reason 2 outlines the risks of ignoring these genetic factors and highlights that poor genetic health can become the single biggest threat to species persistence. Fortunately, “genetic management” provides large benefits for individual fitness, population growth, and species resilience, as described in Reason 3. Reason 4 addresses the long-held perception that genetically distinct populations need to be managed separately, and summarizes why current evidence generally suggests otherwise. In Reason 5, we show that “genetic management” is well established to be cost-effective, thereby busting the common misconception that genetic tools are too costly. Reasons 6 and 7 deal with practicalities, showing that sample collection for genetic health assessments can have low ethical and environmental impacts, and that genetic health assessments do not require the most recent, cutting-edge, or complex genetic techniques.

**Table 1.** Seven reasons why threatened species managers must consider genetic health.

No.	Reason
**1**	Threatened species are likely to have poor genetic health.
**2**	Poor genetic health substantially increases extinction risk.
**3**	Management actions to improve genetic health are simple, reliable, and evidence-based.
**4**	Arguments for keeping populations separated need to be re-assessed in light of current evidence.
**5**	Genetic management can be a money- and time-saving conservation tool.
**6**	Sample collection for genetic health assessments can have low ethical and environmental impacts.
**7**	Genetic health assessments do not require the most recent, most expensive, cutting-edge technologies.

## Reason 1: Threatened species are likely to have poor genetic health

Threatened species must be assessed for genetic health risks. Losses of genetic health occur in small, fragmented populations, and threatened species typically have small and/or declining populations (Frankham et al. 2017, Allendorf et al. 2022). In a meta-analysis of 170 threatened species, Spielman and colleagues (2004) found that threatened species had, on average, 35% lower genetic diversity compared to related non-threatened species. As outlined in Reason 2, loss of genetic diversity and the resulting increase in inbreeding reduces individual fitness and contributes to extinction risk. This is even more the case in many threatened species due to increased genetic relatedness within populations, where “population” is defined as a group of individuals of the same species that live close enough together that mating is possible (Waples and Gaggiotti 2006).

Most species will respond similarly to poor genetic health, including land plants, invertebrates, amphibians, reptiles, mammals, and birds, provided they have a similar mating system (e.g., naturally outbreeding, not self-fertilizing) and mode of inheritance (e.g., diploid rather than polyploid, haplodiploid, or asexual; Roff 1997, Crnokrak and Roff 1999, Keller and Waller 2002, Angeloni et al. 2011, Frankham et al. 2017, 2019). Hence, generalizations from other well-researched species can reasonably be used to guide decision-making with respect

to the genetic health of threatened species (e.g., Frankham et al. 2014, 2017, Kriesner et al. 2019). For discussions of genetic health in species with diverse mating systems and modes of inheritance, see Dudash and Murren (2008), Severns and Liston (2008), Zayed (2009), Weeks and colleagues (2011), and Frankham and colleagues (2026).

It is not advisable to wait until the consequences of genetic health (i.e., inbreeding depression or the inability to adapt) are detected in a threatened species before acting. Even in severe cases, inbreeding depression cannot easily be detected without a rigorous study design, sufficient statistical power, and data on total fitness (Keller and Waller 2002, Harrisson et al. 2019, Ralls et al. 2020). Detection can even be difficult for species in breeding programs with pedigrees (Kalinowski and Hedrick 1999). Hence, genetic health assessments should be conducted based on likely risk, and detailed risk assessment guidelines are presented by Frankham and colleagues (2017, 2019; also see Pavlova et al. 2025). The presence of small, isolated populations (e.g., fewer than 1000 adult individuals) can serve as a simple trigger for conservation managers to seek expert guidance for assessing genetic health and beginning genetic management (Frankham et al. 2014). Most threatened species globally will need genetic management to avoid extinction of at least some of their populations (Frankham et al. 2017, Ralls et al. 2018).

## Reason 2: Poor genetic health substantially increases extinction risk

Poor genetic health must be considered alongside habitat loss, invasive species, disease, and climate change as a major threat to biodiversity. This is because it severely affects fitness, adaptability, vulnerability to other threats, and extinction risk as a direct result of an increase in inbreeding and a loss of genetic diversity (Frankham et al. 2017).

**Figure 1.**
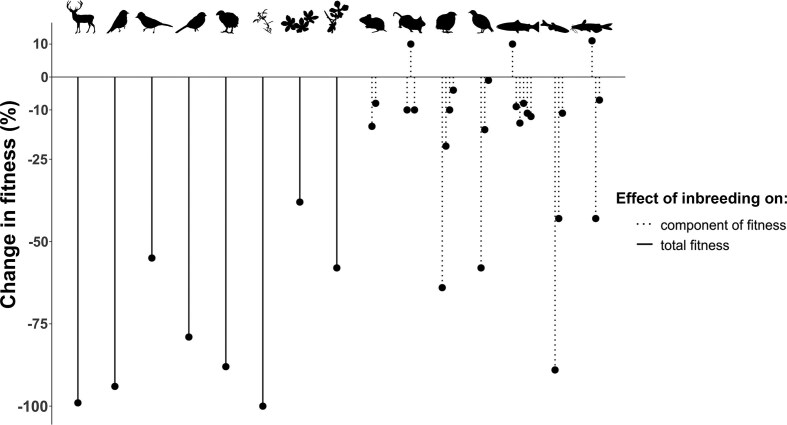
Examples of the effect of inbreeding depression on fitness traits in wild species of animals and plants, as reviewed in Frankham and colleagues (2010, 2017). All examples are measured as a percentage difference in a fitness trait between inbred (to the level found in the offspring of a mating between full siblings) and outbred offspring reared in similar environments. “Total fitness” refers to estimates of the total number of adult offspring an individual produces in their lifetime. “Component of fitness” refers to any life history trait that contributes to total fitness, such as fertility or survival to a particular age. Across these examples, estimates of inbreeding depression in total fitness are on average greater than those for individual fitness components, following the expected pattern that component measures underestimate the full impact of inbreeding on overall fitness (Huisman et al. 2016, Frankham et al. 2017, Harrisson et al. 2019). Further, in many cases, the degree of inbreeding depression in this figure is likely an underestimate because (a) studies on birds have not accounted for extra-pair paternity, and (b) studies on plants (and the Collared Flycatcher) did not include the contributions of maternal inbreeding to the reduction in total fitness (Frankham et al. 2017). Studies of total fitness as reviewed in Frankham and colleagues (2017), from left to right: Red Deer (*C. elaphus*; Huisman et al. 2016), Collared Flycatcher (*F. albicollis*; Kruuk 2004), Great Tit (*Parus major*; Szulkin et al. 2007), Song Sparrow (*M. melodia*; Keller 1998), South Island Takahē (*P. hochstetteri*; Grueber et al. 2010), Deerhorn Clarkia (*C. pulchella*; Newman and Pilson 1997), Rose Pink Plant (*Sabatia angularis*; Dudash 1990), and Wild Radish (*Raphanus raphanistrum sativus*; Nason and Ellstrand 1995). Studies of components of fitness from Abplanalp (1990) and Roff (1997) as reviewed in Frankham and colleagues (2010), from left to right: Prairie Deer Mouse (*Peromyscus maniculatus bairdii*; litter size and survival to weaning; Hill 1974, Haigh 1983), House Mouse (*Mus musculus*; litter size, body weight, and nesting behavior; Lynch 1977), Japanese Quail (*Coturnix japonica*; reproduction and survival, fertility, survival 0–5 weeks, and body weight; Sittmann et al. 1966), Chukar Partridge (*Alectoris chukar*; reproduction and survival, egg production, and body weight; Abplanalp 1990), Rainbow Trout [*Oncorhynchus mykiss*; hatchability (*n* = 3), fry survival (*n* = 2), and weight at 150 days; Kincaid 1976a, 1976b, Gjerde et al. 1983]; Zebra Fish (*Danio rerio*; hatchability, survival to 30 days, and length at 30 days; Mrakovčič and Haley 1979); Channel Catfish (*Ictalurus punctatus*; hatchability, body weight at 4 weeks, and body weight at 12 weeks; Bondari and Dunham 1987).

Just as in Humans (Ceballos and Álvarez 2013), breeding between related individuals (inbreeding) leads to substantial decreases in fitness, as measured by impacts on survival, testes development and sperm quality in males, female fecundity, cognition, and resistance to infectious diseases (e.g., Roelke et al. 1993, Coltman et al. 1999, Fitzpatrick and Evans 2009, Colpitts et al. 2024, Gavriilidi and Van Linden 2024). Although individual abnormalities are easily observable in inbred individuals, the full consequence of inbreeding depression is a decrease in reproductive output over an individual’s entire lifetime (“total fitness”; Huisman et al. 2016, Harrisson et al. 2019). These fitness decreases are typically substantial (see figure 1, e.g.), with inbreeding experienced by the offspring of a mating between full siblings resulting in >70% decrease in the total fitness of Song Sparrow (*Melospiza melodia*) and South Island Takahē (*Porphyrio hochstetteri*) and >90% decrease in the total fitness of Red Deer (*Cervus elaphus*), Collared Flycatcher (*Ficedula albicollis*), and Deerhorn Clarkia plant (*Clarkia pulchella*).

The loss of individual fitness due to inbreeding causes a reduction in population growth that increases extinction risk (Frankham 2005, O’Grady et al. 2006). A well-studied field example is that of the reintroduced populations of threatened Glanville Fritillary Butterfly (*Melitaea cinxia*), where two-thirds of the non-inbred butterfly populations—but none of the inbred populations—survived through their first winter (Nieminen et al. 2001). Given that reproductive output and extinction risk are the metrics most relevant to managers of threatened species, inbreeding must be high on the priority list of conservation risks to manage and mitigate.

In addition to the impacts of inbreeding, a loss of genetic diversity heightens extinction risk by reducing a population’s resilience to challenges such as heat waves, cold snaps, droughts, floods, habitat disturbance, new disease strains, or the introduction of invasive species (Frankham et al. 2019, Allendorf et al. 2022). In genetically diverse populations, individuals show variation in their ability to respond to different environmental challenges, with those better suited to the new environment surviving, meaning that the population can persist. This is the process of adaptation through natural selection. In contrast, low genetic diversity reduces the range of conditions in which individuals within a population can tolerate and persist (Frankham 2005). In a laboratory experiment, genetically diverse populations of Least Killifish (*Heterandria formosa*) were able to adapt to high temperatures over four generations, but bottlenecked populations with lower genetic diversity showed a reduced ability to adapt (Klerks et al. 2019). Similarly, coastal populations of Golden Kelp (*Ecklonia radiata*) with high genetic diversity were more likely to survive a marine heat wave than lower diversity populations (Wernberg et al. 2018). Hence, in the long term (i.e., over generations), genetic diversity is a fundamental requirement for species to adapt to new and challenging conditions through natural selection (Kardos et al. 2021).

The contribution of genetic factors to extinction risk is not a new idea and has been extensively demonstrated across laboratory, field-based, and simulation studies (Keller and Waller 2002, Frankham 2005). As such, poor genetic health must be thoroughly assessed along with other likely threats to a species, such as habitat loss, invasive species, and bushfires (Ralls et al. 2018).

## Reason 3: Management actions to improve genetic health are simple, reliable, and evidence-based

Poor genetic health is the detrimental outcome of increased inbreeding and loss of genetic diversity in small, isolated populations (Reason 2). In populations with poor genetic health, the practice of “augmented gene flow” can restore genetic health simply and rapidly (Weeks et al. 2011, Frankham et al. 2017). Augmented gene flow involves moving unrelated individuals into a potentially inbred group, whether that is into a geographically isolated family group, or into an entirely different population (Weeks et al. 2011). Restoring effective movement corridors also has the same effect, but over longer time scales. When successful, augmented gene flow results in an increase in population fitness known as a “genetic rescue” (Weeks et al. 2011). The benefit can be maximized by sourcing individuals that are genetically diverse and with very low genetic similarity to the target group (as measured by kinship), such as from a larger, unrelated population. Sourcing individuals without regard for their contribution to genetic health (e.g., using individuals from poorly managed breeding programs) will not achieve the same benefit, but might be better than doing nothing if there are no other options (Frankham 2015, Grueber 2017).

Augmented gene flow does not require the movement of large numbers of individuals; rather, the regular movement of small numbers of individuals (e.g., tens of adults per generation) between populations can be ideal (Weeks et al. 2011, Frankham et al. 2019). Even once-off movements of small numbers of individuals can have measurable improvements to genetic health. Weeks and colleagues (2017) report significant short-term improvements to fitness and genetic diversity in a population of Mountain Pygmy Possum (*Burramys parvus*) after the introduction of six unrelated males. Similarly, Florida Panther (*Puma concolor coryi*) saw improvements to juvenile survival, genetic diversity, inbreeding, and population growth after the introduction of only eight unrelated adults (Johnson et al. 2010, Hostetler et al. 2010, 2012). Even a single immigrant can, to some degree, reduce inbreeding and benefit reproductive fitness in a small, isolated population (e.g., Spielman and Frankham 1992). Published recommendations exist for determining the optimum number of individuals to use in augmented gene flow, which can vary based on the size of the source and recipient populations, the severity of inbreeding, and the desired duration of impact (Weeks et al. 2011, Frankham et al. 2019, Pavlova et al. 2025).

Research into augmented gene flow shows that it is a very reliable management practice in a range of scenarios and can cause a large and persistent genetic rescue (Grueber 2017). A meta-analysis by Frankham (2015) assessed cases of augmented gene flow into inbred populations that had a low risk of outbreeding depression (based on an outbreeding depression screen from Frankham et al. (Frankham et al. 2011)). The meta-analysis showed that 93% of 156 applications of augmented gene flow successfully increased fitness, with a median increase of 162.5% in total fitness for cases of wild populations (Frankham 2015). In contrast, the cost of not performing a necessary augmented gene flow can be catastrophic, with studies showing that small, inbred populations have lower population growth and a heightened extinction risk compared to those that receive augmented gene flow (Westemeier et al. 1998, Weeks et al. 2011, Harrisson et al. 2016, 2017, Trask et al. 2019). We acknowledge that genetic health is only one aspect of a threatened species recovery program. However, management strategies that reduce ecological threats and improve population size may have only a limited effect on extinction risk if not applied simultaneously with augmented gene flow (e.g., Westemeier et al. 1998, Hufbauer et al. 2015, Trask et al. 2019, Encinas-Viso et al. 2024). Augmented gene flow therefore must be considered as a top priority tool by conservation managers of threatened species (Ralls et al. 2018). Early implementation is recommended, with the aim of creating resilient multi-source populations, with sufficient gene flow either augmented (Pavlova et al. 2025) and/or achieved through other recovery actions such as increased habitat connectivity or threat reduction.

## Reason 4: Arguments for keeping populations separated need to be re-assessed in light of current evidence

There is compelling evidence supporting the importance of genetic health (see Reasons 1 and 2) and the benefits of augmented gene flow between populations (see Reason 3). Once conservation managers identify that a population or species might benefit from genetic management, experts can support them in accurately determining the best course of action and appraising likely outcomes. Furthermore, simple decision-making trees and risk assessment tools have been developed that support conservation managers in determining which species might benefit (e.g., Weeks et al. 2011, Hoffman et al. 2015, Frankham et al. 2019, Kriesner et al. 2019, Fitzpatrick et al. 2023). However, several barriers to evidence-based genetic management still exist based primarily on the perception that it is bad to mix different populations, especially if they are believed to be ecologically or evolutionarily “different” (Love Stowell et al. 2017, Ralls et al. 2018, Liddell et al. 2021). Much of this resistance can be linked to a few simple misconceptions that are not supported by current evidence. For example, “outbreeding depression” is a loss of fitness caused by mixing individuals that have a high level of evolutionary divergence (e.g., between different species). Although outbreeding depression is real, the concept is often misapplied, preventing evidence-based assessments that would have recommended augmented gene flow (Ralls et al. 2018, Liddell et al. 2021). We briefly summarize this and other misconceptions in table 2. Ultimately, using a simple screen for outbreeding depression—as proposed by Frankham and colleagues (2011) and tested by Frankham (2015)—will ensure the benefits of increased fitness from augmented gene flow will outweigh the risks.

**Table 2.** Some typical misconceptions and their corresponding clarifications regarding mixing populations for the purpose of augmented gene flow.

Misconception	Clarification
**Augmented gene flow will swamp important local adaptation** (Weeks et al. 2016, Love Stowell et al. 2017).	**Augmented gene flow is more likely to maintain local adaptation than keeping the population isolated** (Fitzpatrick et al. 2020, Zilko et al. 2021). Careful planning is required to ensure the amount of gene flow is controlled and monitored. Meanwhile, backcrossing and natural selection ensure that beneficial traits are maintained in any particular environment (Frankham et al. 2017, Weeks et al. 2017).
**Augmented gene flow will erode a population’s uniqueness or distinctiveness** (Weeks et al. 2016, Love Stowell et al. 2017).	**The idea that a population’s uniqueness makes it more “natural” or “pure” is not biologically realistic** (Love Stowell et al. 2017). Genetic uniqueness can be an artifact of inbreeding itself as well as anthropogenic isolation (Weeks et al. 2017). Protecting a population’s distinctiveness may directly result in extinction (Frankham et al. 2017, Weeks et al. 2017).
**Hybridization between lineages, sub-species, or evolutionary significant units (ESUs) to alter their genetics is “unnatural”** (Love Stowell et al. 2017, Zander et al. 2021).	**Within-species units (e.g., sub-species, lineages, and ESUs) can be based on inconsistent or misleading definitions** (Frankham et al. 2017). Hybridization between these units, and even between species, occurs frequently in nature (Payseur and Rieseburg 2016, Ottenburghs 2019) and can be essential for the transfer of adaptive alleles in wild populations (Hedrick 2013, Suarez-Gonzalez et al. 2018, Giska et al. 2019). In conservation settings, hybridization between within-species units can reverse poor genetic health (e.g., Pavlova et al. 2023). Poor genetic health is itself often unnatural as it is typically a result of human-caused isolation and habitat fragmentation (Frankham et al. 2017).
**Outbreeding depression is a major risk of mixing populations** (Ralls et al. 2018, Liddell et al. 2021).	**The occurrence of outbreeding depression is predictable, and when present is often still outweighed by the benefits of restoring genetic health** (Frankham et al. 2011, Frankham 2015). Augmented gene flow is mainly recommended for recently isolated populations (less than 500 years; Frankham et al. 2011) to lower this risk. But where necessary, experimental attempts at augmented gene flow have been successful (i.e., resulted in genetic rescue) between lineages that diverged 10,000 years ago (Wells et al. 2019), 20,000 years ago (Weeks et al. 2017), and even 50,000 years ago (Pavlova et al. 2023).
**The survival of some small, isolated populations proves that they can be “purged” of harmful genetic load, that inbreeding depression does not exist in many cases, or that augmented gene flow from genetically diverse populations is ill-advised** (Ralls et al. 2020).	**The few observations of these situations are a result of “survivorship bias.” Poor genetic health increases extinction risk, but the temporary survival of a few populations out of many is always possible** (Reed 2010, Ralls et al. 2020). There is little evidence that purging is effective enough to fully eliminate dangerous levels of inbreeding depression (Leberg and Firmin 2008, Grossen et al. 2020, Ralls et al. 2020, Kardos et al. 2023). Furthermore, inbreeding depression is difficult to detect in affected populations without a rigorous study design, sufficient statistical power, and data on total fitness (Ralls et al. 2020). It is even more difficult to detect in captive populations, where conditions are benign even for inbred individuals (Frankham 2015). Inbreeding depression can be assumed to be present for any sexually reproducing species that has become inbred (Ralls et al. 2020).

## Reason 5: Genetic management can be a money- and time-saving conservation tool

A lack of resources hampers threatened species and biodiversity management (Craigie and Pressey 2022). As a result, a lack of funding is considered a huge barrier to the implementation of genetic management (Cook and Sgro 2018). Yet genetic health assessments are not as expensive as generally perceived, and augmented gene flow can yield highly cost-effective conservation outcomes compared to other recovery actions (Hufbauer et al. 2015, Weeks et al. 2017, Ralls et al. 2018, Trask et al. 2019). This is because of the following:

- Unlike other more common forms of conservation management, the direct benefits to fitness of improving genetic health are large, predictable, immediate, and persistent (see Reason 3; Frankham 2015, Ralls et al. 2018, Frankham et al. 2019). Augmented gene flow also has low risk provided the likelihood of outbreeding depression is assessed using evidence-based guidelines (see Reason 4; Frankham et al. 2011, Frankham 2015).
- Conservation managers can export many of the costs and workload of genetic health assessments by collaborating with university-based researchers, who are proficient at obtaining grants and can take on much of the analysis in-kind as a collaboration or as training for research students (Hogg et al. 2022, Bertola et al. 2023).
- Genetic data can provide information, such as kinship, barriers to connectivity, and effective population size, that would be time-consuming, difficult, or impossible to obtain from non-genetic approaches (Kardos et al. 2016, Brandies et al. 2019, Waples 2022). Meanwhile, genetic sampling of individuals can (and should) take place during other conservation activities, reducing the costs of double-handling.
- Genetics-informed conservation actions can be more efficient at achieving pre-existing conservation goals. For example, genetic studies can reveal where habitat restoration would be most valuable, and whether the restoration provided connectivity (Thomas et al. 2022, Bertola et al. 2023), the optimal breeding strategy for a captive population (Harrisson et al. 2019, Scott et al. 2020), which captive-born individuals to use in a reintroduction (Magro Moraes et al. 2023), and whether translocated individuals have actually bred with the recipient population (Taylor et al. 2024).
- There are many ways to begin genetic management with limited or no genetic data (Frankham et al. 2019, O’Brien et al. 2022), even if based simply on identifying small, isolated populations (e.g., fewer than 1000 adult individuals). At the very least, this can involve a triage of priority species before allocating available resources (Kriesner et al. 2019, O’Brien et al. 2022, Fitzpatrick et al. 2023). National and international repositories of pre-existing genetic data are also available (Hogg et al. 2024).

Conservation managers can therefore look at genetic health assessments and genetic management as a low-cost opportunity. To assist with this, it is important that researchers publish standardized cost-effectiveness analyses of various management actions, so their benefits and affordability can be clearly assessed by managers (Cook et al. 2017).

## Reason 6: Sample collection for genetic health assessments can have low ethical and environmental impacts

**Figure 2.**
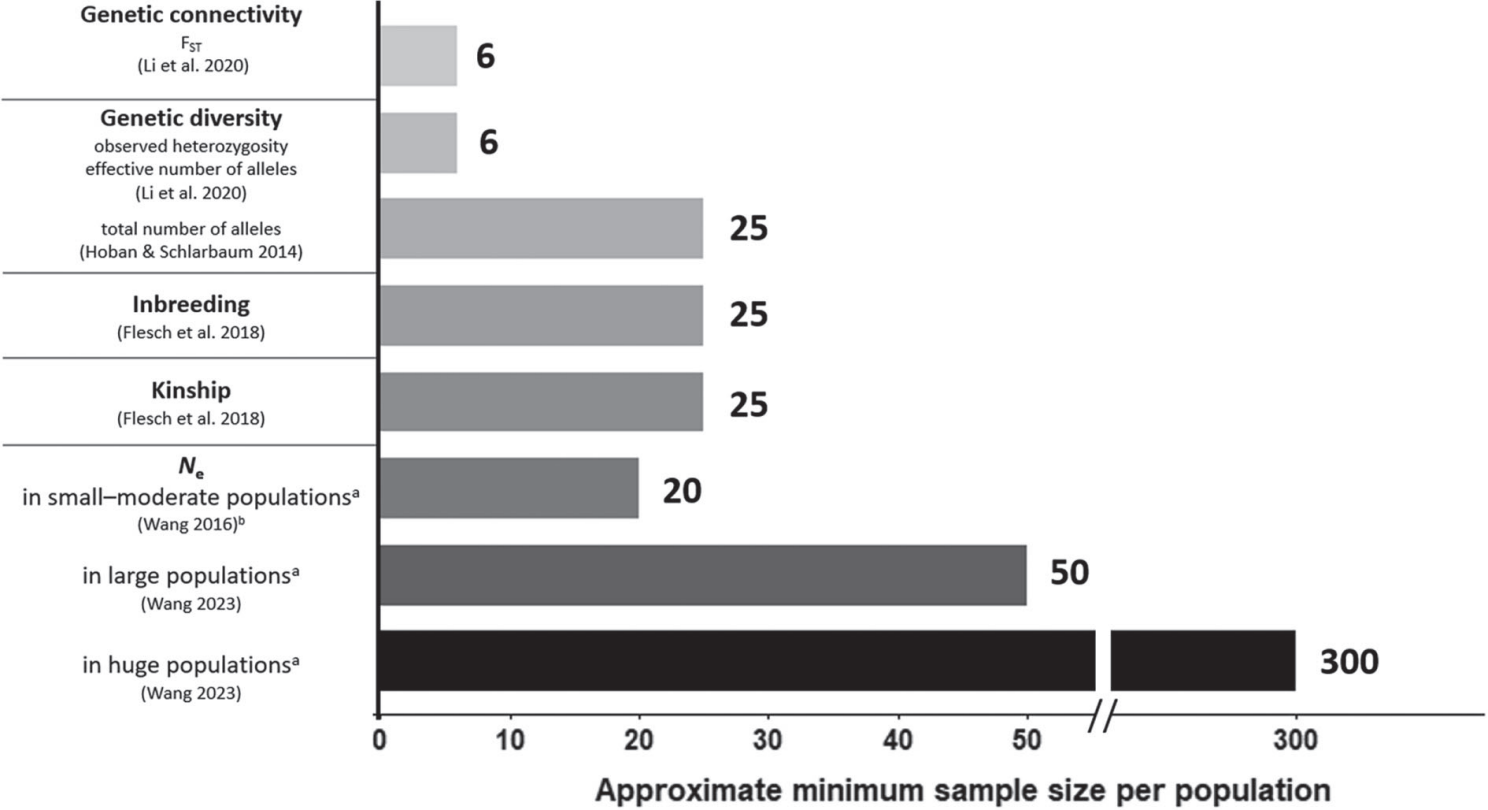
Approximate minimum sample sizes per population for estimating different measures of genetic health. These values are based on the cited power analyses and intended only as a rough guide. These recommendations are made with the assumption that a dataset of thousands of genetic markers (e.g., SNPs) is available. A range of methods with different requirements can exist for each measurement, and these are constantly being developed. Studies cited: Hoban and Schlarbaum (2014), Wang (2016, 2023), Flesch and colleagues (2018), and Li and colleagues (2020).^a^Here, small–moderate refers to *N*_e_ ≲ 500 (*N*_c_ ≲ 5000), large refers to *N*_e_ ≈ 1000 (*N*_c_ ≈ 10,000), and huge refers to *N*_e_ ≈ 100,000 (*N*_c_ ≈ 1,000,000), with the assumption that *N*_e_ is one-tenth of the census size (*N*_c_). In reality, the *N*_e_/*N*_c_ ratio can vary widely (see box 1).^b^Power analysis conducted using only 20 unlinked loci, but this recommendation is also sensible for a dataset containing thousands of SNPs.

Conservation managers have valid concerns about the impacts of collecting samples for genetic health assessments; in particular, the potential harm to sampled individuals (Soulsbury et al. 2020). An important, overarching consideration regarding this concern is the minimum number of samples required for informative estimates of genetic health. To give readers an indication of approximate minimum sample sizes per population, figure 2 summarizes a limited number of studies focused on sample size determination. Importantly, even small sample sizes (e.g., six individuals per population) can give accurate estimates of heterozygosity. Other key genetic health metrics require larger sample sizes, with estimates becoming more accurate at around sample sizes of 20–25 individuals per population.

In terms of obtaining samples, there are two important considerations. First, many museums now have large frozen tissue collections that can form the basis of genetic health assessments. Second, progress in technology and practice is lowering the ethical impact of genetic sampling on animals through a reduction in the amount of tissue required and consequently how that might be obtained. We focus on this here. For many genetic datasets [including single nucleotide polymorphisms (SNPs); see Reason 7 for a discussion of the types of genetic datasets], it is now possible to use very small tissue samples (less than 20 mg), rather than larger samples (e.g., toe-clipping, large biopsies, or blood samples) that involve greater physiological stress (Langkilde and Shine 2006, Narayan et al. 2011, Fisher et al. 2013). Ample DNA can be obtained non-lethally from samples as small as a fraction of a frog toe pad (e.g., Bertola et al. 2023), the tip of a tadpole tail (Othman et al. 2020), hair roots and saliva of a mammal (e.g., Colpitts et al. 2024), the feathers of a bird (e.g., Doyle et al. 2016), and the leg of an insect (e.g., Kozlov et al. 2017). Suitable DNA can also be obtained from larvae, feces, hair, buccal swabs, and skin mucus swabs (e.g., Scheet et al. 2012, Othman et al. 2020, Schmidt et al. 2020, Lefort et al. 2022, Massault et al. 2022).

The development of a reference genome (which is not required for genetic health assessments, see Reason 7) sometimes requires different sampling considerations in order to obtain high-quality DNA, such as flash-freezing tissue, storage at or below −80°C, and obtaining nucleated blood or organ tissue, which may require the death of an individual (Brandies et al. 2019, Dodge et al. 2023, Howard et al. 2025). Fortunately, this requirement is easing as the technology develops (Howard et al. 2025).

Finally, it is helpful to recognize that much greater scrutiny is often given to tissue sampling for genetic studies than to the ethical impacts of routine capture and handling (e.g., for survey, monitoring, mark-recapture, disease surveillance). The impacts of trapping, handling, confining, or removing an animal from its environment for even a short time are often underestimated and can be equivalent to or even greater than genetic sampling itself (Langkilde and Shine 2006, Fisher et al. 2013, Soulsbury et al. 2020). However, these methods (e.g., mist netting, harp, box, pitfall, or funnel trapping) are typically deemed acceptable because they have long been used and are considered standard practice in wildlife work (Soulsbury et al. 2020). With this in mind, ethics committees and permit granting agencies should consider requesting genetic samples when researchers: (a) plan to handle threatened or near-threatened species; (b) expect these species as bycatch (i.e., expected to be caught in a trap intended for another species); or (c) will access sensitive, remote ecosystems. This would increase the amount of valuable information obtained from each animal handled and from each field trip, reducing double-handling or re-using of animals in future genetics projects. A major benefit of this proactive genetic sampling approach is the establishment of baselines for genetic monitoring prior to unexpected range contractions or population declines. Sampling must ensure that metadata are comprehensive and securely retained for future reference.

In a few cases, when all of the above considerations have been made, genetic sampling may still be considered too risky or impactful to undertake at the present time. There are still beneficial genetic management actions that can be made with limited or no genetic data (Frankham et al. 2019, Kriesner et al. 2019, O’Brien et al. 2022). Nevertheless, given the many unknowns, it is usually preferable to have some genetic data.

## Reason 7: Genetic health assessments do not require the most recent, most expensive, cutting-edge technologies

Whole genome sequencing is a comprehensive view of an organism’s genetic code and is often held up as the “gold standard” for conservation genomic research, particularly in the case of “reference genomes,” which support numerous other approaches (Brandies et al. 2019, Wright et al. 2020). However, the technical expertise, time, and funding required for these studies put whole genome sequencing out of reach for most threatened species (Shafer et al. 2015, Wright et al. 2020, Hogg et al. 2022). Using 2019 costs, whole genome resequencing projects typically cost more than USD$644 per sample (Brandies et al. 2019), whereas a single reference genome of average size may cost more than USD$20,000 to develop (Brandies et al. 2019). In addition, in animal studies, the death of an animal may be required to provide enough high-quality DNA (e.g., Dodge et al. 2023; see Reason 6). However, whole genome sequencing is not essential for most conservation genetics questions.

Although genetic assessments based on just one, or a few, loci or genes (e.g., mitochondrial DNA) can be useful for identifying lineages within species, they are not suitable for genetic health assessments or delineating closely related species (Frankham et al. 2017). Older multi-locus techniques, which use only tens of genetic markers or fewer, including microsatellites, continue to provide useful results and should not be dismissed so long as the limitations of the methods are acknowledged from the outset (Sunde et al. 2020, Hauser et al. 2021). Microsatellite datasets can sometimes adequately assess population structure (Hauser et al. 2021), small effective population sizes, and inbreeding in small populations (McLennan et al. 2019). Comprehensive genetic health assessments using microsatellites have been undertaken for Eurasian Otter (*Lutra lutra*; Thomas et al. 2022) and Macquarie Perch (*Macquaria australasica*; Pavlova et al. 2017). However, in general, it is difficult to obtain accurate estimates of inbreeding and relatedness using microsatellite data (Grueber et al. 2010, Taylor 2015, Kardos et al. 2015, 2017), assess moderate–large effective population sizes (Waples 2025), or determine fine-scale genetic structure (Gallego-Garcia et al. 2021).

Fortunately, a middle ground exists in using SNPs datasets generated using “reduced representation methods” [e.g., RADseq (Restriction-site Associated DNA sequencing)]. These contain thousands of genome-wide markers sequenced at relatively little expense compared to whole genomes (using 2019 costs, typically less than USD$64 per sample, plus costs for marker establishment and DNA extraction; Brandies et al. 2019). SNP datasets often outperform microsatellite markers and approach whole genome sequencing in terms of reliability and usefulness for conservation questions (McLennan et al. 2019, Wright et al. 2020, Martchenko and Shafer 2023). SNP-based genetic studies are revolutionizing conservation genetics due to their power and affordability. Typically, SNP datasets require less time and expertise to analyse than a whole genome dataset. They also require far less tissue per sample than whole genome sequencing. Examples where threatened species have received comprehensive genetic health assessments based on thousands of genome-wide SNPs include Lima Leaf-Toed Gecko (*Phyllodactylus sentosus;* Arana et al. 2023); Kuranda Treefrog (*Litoria myola*; Bertola et al. 2023); Alpine She-Oak Skink (*Cyclodomorphus praealtus*; Hartley et al. 2023); Button Wrinklewort Daisy (*Rutidosis leptorrhynchoides*; Rodger et al. 2021); Shiratama Hoshikusa plant (*Eriocaulon nudicuspe*; Saeki and Hirao 2025); Wild Cotton (*Gossypium turneri*; Yescas-Romo et al. 2025); and Northern Bettong (*Bettongia tropica*; Todd et al. 2023). These and other genetic health assessments have led to on-ground management actions.

## What now? How to begin implementing genetics-informed conservation actions in the real world

For readers motivated to learn more or to apply the concepts discussed in this paper, a first step is to contact a scientist with specialist knowledge and experience in conservation genetics, particularly one with local knowledge. Scientists will often have the resources required, are likely to help navigate the costs involved, and will raise additional relevant questions. In addition, they are often highly motivated to collaborate and support conservation efforts. Researcher profiles are typically available on your local research institution’s website (e.g., a university, museum, botanic gardens, or herbarium). However, it is often easier to find relevant experts through existing academic research collaborators. Most conservation departments and organizations will already have relationships with local academics and researchers from various fields, who can help direct you to the most suitable person or team. There are conservation geneticists accessible worldwide, and new experts are being trained all the time.

## Conclusion

Poor genetic health is detrimental to the persistence of plant and animal populations. This is particularly relevant to threatened species because their small, isolated populations are predisposed to losing genetic diversity and becoming inbred. However, these threats are often underestimated. Fortunately, opportunities for straightforward genetic management practices exist, with well-established benefits to individual fitness and population growth. Primarily, this revolves around deliberately introducing unrelated individuals into other groups with low genetic health (i.e., augmented gene flow). Despite perceptions to the contrary, these practices can be low-cost and low-risk when applied using existing recommendations. The assessments required to inform genetic management can have a low ethical impact and be affordable, not requiring large sample sizes or the most expensive techniques. Our aim is that these seven reasons we have provided are motivating to threatened species managers. If they are to prevent further extinctions, threatened species managers must consider genetic health.

## Data Availability

No new data were generated or analysed in support of this research.
